# Are pornography use motivations related to behaviors toward the romantic partner? A dyadic daily diary study

**DOI:** 10.1177/02654075251335813

**Published:** 2025-04-22

**Authors:** Mandy Vasquez, Marie-Ève Daspe, Beáta Bőthe, Sophie Bergeron, Samantha J. Dawson, Marie-Pier Vaillancourt-Morel

**Affiliations:** 1 14847 Université du Québec à Trois-Rivières, Canada; 2 The Interdisciplinary Research Center on Intimate Relationship Problems and Sexual Abuse - CRIPCAS, Canada; 3 5622 Université de Montréal, Canada; 4 University of British Columbia, Canada

**Keywords:** pornography use motivations, positive behaviors, negative behaviors, couple dynamic, daily design

## Abstract

Individuals in couple relationships often use pornography. Previous findings are inconsistent regarding how pornography use may affect couples and tend to focus on overall use and broad retrospective indicators of relationship functioning (e.g., relationship satisfaction). No research has considered the motivations behind pornography use and how they relate to daily dynamics between partners. Yet, the approach-avoidance sexual motivation theory suggests that approach motivations to engage in a sexual activity (e.g., for sexual pleasure) are associated with positive relational outcomes whereas avoidance motivations (e.g., to avoid a conflict) are related to negative relational outcomes. This study bridges these gaps by examining the associations between pornography use motivations and daily positive (e.g., listening to the partner) and negative (e.g., getting angry at the partner) behaviors among couples using a dyadic daily diary design. A convenience sample of 327 couples (50.6% women; *M*_age_ = 31.5) completed daily self-report measures over 35 days. Multilevel actor-partner interdependence models showed that on days when a person used pornography for stress reduction, they reported fewer positive behaviors toward their partner. Similarly, on days when a person used pornography for emotional distraction, they reported fewer positive behaviors and greater negative behaviors toward their partner. On days when a person used pornography for partner-related motivation, they reported greater positive behaviors toward their partner, and on days when a person used pornography for sexual pleasure, they reported fewer negative behaviors toward the partner. For most motivations, on days when a person used pornography, their partner reported fewer positive behaviors toward them that day. Our results are in line with the approach-avoidance sexual motivation theory and support the need to consider motivations underlying pornography use for a better understanding of its associations with same-day couple dynamics.

## Introduction

Pornography use is a frequent sexual activity for individuals currently involved in a romantic relationship. In adult heterosexual couples, between 70.5% and 91.8% of men and between 33.7% and 82.9% of women have used pornography while in a romantic relationship (Kohut et al., 2017; Willoughby et al., 2016). While media outlets assert that pornography use detrimentally affects romantic relationships (Montgomery-Graham et al., 2015), cross-sectional and longitudinal studies have yielded inconsistent conclusions regarding the potential benefits and drawbacks of pornography use for couples. While some studies suggest that pornography use is related to one own’s greater sexual desire, higher relationship satisfaction, and higher sexual and emotional intimacy (Huntington et al., 2021; Willoughby & Leonhardt, 2020), others studies have reported that it is related to lower sexual interest in the partner, lower relationship and sexual satisfaction, and higher odds of intimate partner violence (Kohut et al., 2017; Vasquez et al., 2024; Willoughby et al., 2020).

To address these mixed findings, a recent organizing framework around relational pornography scholarship highlighted the need to consider the context surrounding pornography use, including the motivations underlying this use (Campbell & Kohut, 2017). Motivations underlying a behavior are individual characteristics that can explain in which context that activity is associated with positive or negative outcomes, thus could help untangle past conflicting findings (Bőthe et al., 2022). Moreover, previous studies have predominantly focused on broad retrospective indicators of relationship functioning (e.g., relationship satisfaction; Willoughby & Leonhardt, 2020), neglecting to explore how pornography use may be associated with day-to-day dynamics between partners, including both positive and negative behaviors. Given that dissatisfaction with a partner’s behaviors is a common issue addressed in couple therapy (e.g., few positive or pleasant behaviors, too many negative or unpleasant behaviors; Labonté et al., 2022), it is important to examine whether pornography use might be related to variations in daily positive and negative behaviors expressed between partners. Although daily diaries cannot establish causality, they can offer insights into the short-term correlates of pornography use for couple dynamics on a given day, or even the next day, and enhance our understanding of its potential positive and negative repercussions on both partners. The current study examined the associations between daily pornography use motivations and positive and negative behaviors among adult couples from the community using a dyadic daily diary design.

### Pornography use and couples

A growing body of research has examined the associations between pornography use frequency and romantic relationship functioning. Quantitative cross-sectional and longitudinal intra-individual studies reported mixed findings but overall, results showed that men’s higher frequency of solitary pornography use was related to lower relationship adjustment, commitment, emotional intimacy, and sexual dysfunction, whereas women’s solitary pornography use was associated with higher emotional intimacy, relationship adjustment, and lower sexual dysfunction (Bőthe, Tóth-Király, Griffiths, et al., 2021; Huntington et al., 2021). Dyadic retrospective studies have also shown that men’s solitary pornography use was associated with their partner’s higher intimate partner violence perpetration and lower relationship satisfaction and sexual desire, whereas women’s pornography use was not significantly related to their partner’s relationship satisfaction but was related to their partner’s higher sexual desire (Jongsma & Timmons Fritz, 2021; Vaillancourt-Morel et al., 2020; Willoughby & Leonhardt, 2020).

However, most studies assessed overall pornography use and broad indicators of relationship functioning (e.g., relationship satisfaction) using retrospective reports over a vague period of time (e.g., four or twenty months; Huntington et al., 2021; Jongsma & Timmons Fritz, 2021) which may be biased, have low ecological validity, and preclude the examination of whether pornography use is related to the same day, or even next day, couple dynamic (Vaillancourt-Morel et al., 2020). Thus, even if past studies suggest that pornography use may be related to the couple interactions (e.g., related to positive communication between partners or to violent behaviors; Jongsma & Timmons Fritz, 2021; Vasquez et al., 2024; Willoughby & Leonhardt, 2020), there is a relative lack of knowledge on whether pornography use may be related to the day-to-day couple behaviors, including both positive and negative behaviors expressed toward the partner.

One daily diary study on pornography use among couples showed that a person’s pornography use on one day was not significantly associated with their own or their partner’s relationship satisfaction on the same or the next day (Vaillancourt-Morel et al., 2020). However, when an individual’s solitary pornography use was unknown by their partner, they reported lower relationship satisfaction and intimacy on the same day (Vaillancourt-Morel et al., 2023). Nevertheless, these broad outcomes give little information on the association between pornography use and positive and negative indicators of day-to-day couple interactions. Moreover, although theoretical models (e.g., Antecedent-Context-Effect-model - ACE; Campbell & Kohut, 2017) have highlighted the need to consider the context surrounding pornography use to better understand the possible positive or negative impacts on romantic relationships, most past studies only examined frequency of pornography use. Yet, the motivations behind pornography use could help untangle in which context it is related to positive or negative couple dynamics.

### Pornography use motivations

Individuals use pornography for a wide variety of motivations including out of curiosity, to avoid boredom, to supress or distract from painful emotional states, to reduce stress, because they are sexually dissatisfied, to explore what arouses them sexually, to stimulate fantasy, as a sexual activity with their partner, and most commonly, for sexual pleasure (Bőthe, Tóth-Király, Bella, et al., 2021; Paul & Shim, 2008). In general, men are more likely than women to use pornography as a sexual activity with their romantic partner or with their friends (e.g., as an activity at a bachelor party), regulate their mood (e.g., emotional distraction/suppression, stress reduction, boredom avoidance), for sexual pleasure, out of habit, to stimulate fantasies, and because they are not satisfied sexually (Bőthe, Tóth-Király, Bella, et al., 2021; Paul & Shim, 2008). However, men and women do not differ when it comes to using pornography out of curiosity and to explore what arouses them (Bőthe, Tóth-Király, Bella, et al., 2021).

The approach-avoidance sexual motivations theory (Cooper et al., 2011; Impett et al., 2005, 2008) suggests that when a person engages in partnered sexual activities for approach motives (e.g., to promote one own’s sexual pleasure), they experience positive relationship outcomes (e.g., greater relationship satisfaction, closeness, positive affect, and lower conflicts), whereas when they engage in partnered sexual activities for avoidance motives (e.g., to avoid a conflict), they experience less relationship satisfaction and closeness, and greater negative affect and conflicts. Overall, in regard to pornography use motivations, using pornography for fantasy, emotional distraction/suppression, and for stress reduction motivations is usually related to negative individual outcomes such as more problematic use of pornography (e.g., compulsive use, distress; Bőthe et al., 2024), whereas using pornography with the partner is related to more positive outcomes including greater sexual and emotional intimacy, and sexual satisfaction (Huntington et al., 2021; Willoughby & Leonhardt, 2020).

The only cross-sectional study that examined whether pornography use motivations were related to couple’s functioning specifically focused on sexual wellbeing (Bőthe et al., 2022). Mostly in line with the approach-avoidance sexual motivations theory (Cooper et al., 2011; Impett et al., 2005), results showed that among 259 adult mixed-sex/gender couples, men using pornography for sexual curiosity motivation (approach) reported higher partnered sexual frequency, greater sexual satisfaction and function, and lower sexual distress (Bőthe et al., 2022). However, men using pornography for emotional avoidance motivation (avoidance) reported lower sexual function and greater sexual distress, while women using pornography for sexual pleasure motivation (approach) reported lower partnered sexual frequency. None of the pornography use motivations were significantly related to women’s and their partner’s sexual well-being (Bőthe et al., 2022). This cross-sectional study highlights the importance of pornography use motivations to better understand sexual outcomes in couples as some approach motivations are related to positive sexual outcomes while avoidance motivations are associated with less optimal ones. As individuals with higher avoidance motivations are thought to be responsive to threat, predisposed to experience negative affect, and respond in avoidant or fearful ways (Carver & White, 1994; Gray, 1970), using pornography for avoidance motivations might lead to negative couple dynamics including more negative behaviors and less positive one. On the other side, it is proposed that individuals with higher approach motivations have a greater responsiveness to reward cues and are predisposed to experience positive affect and engage in reward-seeking behaviors (Carver & White, 1994). Thus, using pornography for approach motivations might lead to positive couple interactions including fewer negative behaviors and more positive one. Yet, to our knowledge, pornography use motivations have never been studied in association with positive and negative behaviors toward the partner or using daily diaries, which could help to understand potential short-term outcomes of pornography use on daily couple dynamics. Moreover, as a person’s pornography use motivations may vary from day-to-day, daily diary methods provide a more precise and fine-grained assessment to examine, within an individual, days on which pornography was used for a specific motivation and how it is related to their positive and negative behaviors that day or the next day.

### Current study

The aim of the present study was to examine the associations between daily pornography use motivations and positive and negative behaviors among adult couples using a dyadic daily diary design over 35 days. We hypothesized that on days a person reported using pornography for approach motivations (i.e., sexual pleasure, sexual curiosity, self-exploration, and intimate partner-related), they would report having engaged in more positive behaviors and fewer negative behaviors towards the partner that day. In contrast, on days a person reported using pornography for avoidance motivations (i.e., fantasy, boredom avoidance, lack of sexual satisfaction, emotional distraction, and stress reduction), they would report having engaged in less positive behaviors and more negative behaviors toward the partner that day. As the only dyadic study that have examined the associations between pornography use motivations and sexuality in couples did not find significant partner effects (i.e., how a person’s pornography use motivations are related to their partner outcomes; Bőthe et al., 2022), the associations between a person’s motivations for using pornography one day and their partner’s same day positive and negative behaviors were examined in an exploratory way. Potential differences between cisgender women and cisgender men in the associations were also examined in an exploratory manner. Finally, as repeated daily time points allow for examining whether the associations between today’s pornography use motivations and today’s positive and negative behaviors carries over the next day’s behaviors, we also conducted exploratory lagged daily models.

## Method

### Participants

A convenience sample of 654 participants (*n =* 327 couples) was recruited for this study. This sample included 45.7% (*n* = 299) of cisgender men, 50.6% (*n* = 331) of cisgender women, and 3.4% (*n* = 22) of gender diverse individuals (i.e., transgender, non-binary, genderfluid, multi-gender, genderqueer, agender, another gender). Participants ranged in age from 18 years to 69 years (*M* = 31.51 years, *SD* = 8.42). Most participants defined their sexual orientation as heterosexual (73.7%; *n* = 482), with 8.6% (*n* = 56) identifying as bisexual, 6.3% (*n* = 41) as heteroflexible or homoflexible, 3.5% (*n* = 23) as gay or lesbian, 3.4% (*n* = 22) as pansexual, 2.3% (*n* = 15) as queer, and 2.3% (*n* = 15) reported another sexual orientation (e.g., asexual, demisexual). Most participants (92.2%; *n* = 603) described their ethnic/racial background as White, 1.8% (*n* = 12) as Latin American, 4.0% (*n* = 26) as Asian, 1.7% (*n* = 11) as Indigenous, 0.8% (*n* = 5) as Black, and 3.4% (*n* = 22) reported others ethnic/racial backgrounds (e.g., Arab, Caribbean). As for education, 3.5% (*n =* 23) reported having an elementary school, 7.5% (*n =* 49) a completed high school degree, 10.4% (*n* = 68) a vocational degree, 26.6% (*n* = 174) a college degree, 32.6% (*n* = 213) a bachelor’s degree, 16.4% (*n* = 107) a graduate degree, and 3.1% (*n =* 20) reported reaching others education degrees. Most participants (65.7%; *n* = 430) reported working full or part-time, 20.0% (*n* = 131) were studying, 4.1% (*n* = 27) were on parental leave, 2.6% (*n* = 17) were unemployed, 1.8% (*n* = 12) were homemakers, 1.2% (*n* = 8) were retired, and 4.4% (*n* = 29) reported others principal occupations. Most participants reported an average annual personal income of less than $49,999 CAD (62.5%; *n* = 409), 33.8% (*n* = 221) reported between $50,000 and $99,999 CAD, and 3.7% (*n* = 24) reported more than $100,000 CAD. In regard to their religious identification, 36.5% (*n* = 239) of participants reported being Atheist, 20.3% (*n* = 133) reported having no religious identification, 19.6% (*n* = 128) reported being Catholic, 11.2% (*n* = 73) Agnostic, 6.6% (*n* = 43) Christian, 1.7% (*n* = 11) New Age, Wiccan, or Pagan, and 4.1% (*n* = 27) reported other religious affiliations.

Most participants (87.8%, *n* = 287) formed mixed-gender couples (i.e., cisgender man-cisgender woman), 5.8% (*n* = 19) formed same-gender couples, and 5.8% (*n* = 19) of couples had at least one gender diverse partner. All couples cohabited and 9.2% (*n* = 30 couples) were married. Couples had been together for at least six months to 34.67 years (*M* = 6.32 years, *SD* = 5.88). A total of 56.0% (*n* = 183) of couples had no children, with others reporting between one to five children (*M* = 0.90, *SD* = 1.21).

### Procedure

Data were collected as part of a larger daily diary and longitudinal study among couples (Zephyr Project). This project was advertised as an online study on emotions and sexual and relational well-being of couples. Recruitment occurred between December 2020 and June 2021. Interested participants first completed online screening questions including their contact information and were then contacted by a research assistant for a brief telephone eligibility interview. To be eligible, both partners had to be at least 18 years of age and living together for at least six months. Then, eligible couples independently accessed a unique hyperlink to give their informed consent and completed self-reported questionnaires hosted on Qualtrics Research Suite. Three simple attention-testing questions were distributed within the baseline survey. Participants who failed at least two of these questions were excluded from the study (*n* = 2), and their data were deleted. Each partner received $10 CAD after completing the baseline survey. When both partners had completed the baseline survey, they were contacted by a research assistant to explain the daily diaries’ procedure and set a starting date. Each partner accessed a unique hyperlink received via email or text message each evening to complete a brief survey for 35 consecutive days. Participants were instructed to complete the survey every day before going to sleep. For the daily diaries, compensation was prorated based on how many diaries participants completed, with a maximum of $70 CAD for completing at least 85% of their diaries (30 entries out of 35). All procedures were approved by the Université du Québec à Trois-Rivières’ Institutional Review Board.

Of the 1249 interested couples who started the online screening, 401 were eligible, gave their contact information, and were reached for the telephone eligibility interview. Of these couples, 372 (744 participants) were still eligible and interested to participate after the phone interview. Of the 372 couples who received the baseline survey, 31 dropped out during the baseline survey and two failed at least two out of the three attention-testing questions, resulting in 339 couples for which both partners completed the baseline survey and were invited for the daily diaries. Of the 339 couples, 10 additional couples dropped out before starting the daily diaries or during the first two days and two separated during the daily diaries. Thus, the final sample included 327 couples (96.5%; 654 participants).

### Measures

#### Daily pornography use motivations

We provided the following definition of pornography use to participants (Kohut et al., 2020): “Using pornography means to intentionally look at, read, or listen to: (a) pictures, videos, or films that depict nude individuals or people having sex; or (b) written or audio material that describes nude individuals or people having sex.”. Then, one item was used to assess pornography use in the last 24 hours: “Did you use pornography today?”. If pornography use was reported that day, 12 items assessed nine motivations for using pornography that day. Eleven items were from the Pornography Use Motivations Scale (PUMS; Bőthe, Tóth-Király, Bella, et al., 2021) and asked participants to indicate if they used pornography that day for sexual pleasure (two items, “because it makes masturbation easier”, “to arouse myself sexually”), sexual curiosity (one item, “to learn new things”), fantasy (one item, “because it provides such an experience that would be impossible in real life”), boredom avoidance (one item, “because I want to pass time when I am bored”), lack of sexual satisfaction (one item, “because I am not content with my sexual life”), emotional distraction or suppression (two items, “because it makes me forget my problems”, “to suppress my bad mood”), stress reduction (two items, “because it calms me down”, “because it is one of the ways to relieve stress”), and self-exploration (one item, “because I can find out what turns me on”). Inspired by the Motivations for Internet Pornography Use (Paul & Shim, 2008), one additional item assessed partner-related motivation (one item, “because my partner wanted to watch it together”). Participants could select more than one motivation. Each motivation was then coded as 0 = *no pornography use for this motivation today* and 1 = *pornography use for this motivation today*.

#### Positive behaviors

Positive behaviors toward one’s partner each day were assessed using four items from the measure used in Murray et al. (2003). Participants were asked to what extent the following behaviors occurred today: “I made time to be with my partner”, “I listened to my partner”, “I did something thoughtful for my partner”, and “I was physically affectionate toward my partner”. Items were rated on a seven-point Likert scale ranging from 1 = *Not at all* to 7 = *A lot.* A total score was computed by averaging the four items with higher scores indicating greater positive behaviors that day. In the present study, the McDonald’s omega was .87 and the within-person reliability of change was .79.

#### Negative behaviors

Negative behaviors toward one’s partner each day were assessed using four items from the Hostile Behaviors Scale (HBS; Cui et al., 2005). Participants were asked to what extent the following behaviors occurred on a given day: “Get angry at your partner”, “Criticize your partner or his or her ideas”, “Shout or yell at your partner because you were mad at him or her”, and “Argue with your partner whenever you disagreed about something”. Items were rated on a seven-point Likert scale ranging from 1 = *Not at all* to 7 = *A lot*. A total score was computed by averaging the four items, with higher scores indicating greater negative behaviors that day. In the present study, the McDonald’s omega was .83 and the within person reliability of change was .77.

#### Control variables

Relationship duration was assessed at baseline with one question: “How long have you been in your current romantic relationship?”. Even though partners’ responses were strongly correlated (*r* = .997, *p* < .001), some minor differences naturally occurred. Therefore, a mean score for relationship duration was calculated from both partners’ answers.

Religiosity was measured at baseline using the Religious Engagement Subscale of the Religiosity Inventory (RES; Pennycook et al., 2012) which included three items (e.g., “How important was religion in your daily life?”). Items were rated on various five to seven-point scales ranging from 1 = *irrelevant* or *never* to 5, 6, or 7 = *more than once a week, highest importance,* or *several times a day*. Items are summed to provide a total score ranging from 3 to 18, with higher scores indicating greater religiosity. In the present study, the McDonald’s omega was .83.

Stress related to COVID-19 was assessed at baseline with one question: “In the last three months, how would you rate the amount of stress in your life related to the COVID-19 pandemic?”. Item was rated on a seven-point scale ranging from 1 = *No stress* to 7 = *Extreme stress*.

Masturbation frequency was measured each day with the following item: Did you masturbate alone today (stimulate one’s own genitals for sexual pleasure without a partner)? Item was rated on a seven-point scale ranging from 0 = *No, not at all* to 6 = *Yes, more than five times today*. This item was recoded as 0 = *no masturbation today* and 1 = *masturbation today*.

Partnered sexual activity frequency was assessed each day with one question: “Did you have a sexual activity with your partner today?”. Item was rated on a three-point scale, 0 = *No sexual activity*, 1 = *Yes, and it did not involve intercourse*, and 2 = *Yes, and it involved intercourse*. This item was recoded as 0 = *no sexual activity with the partner today* and 1 = *partnered sexual activity today*. Partners’ reports of sexual activity were highly correlated (*r* = .913, *p* < .001). A mean score for sexual activity frequency was therefore calculated from both partners’ answers.

### Statistical analyses

Descriptive statistics and bivariate correlations were performed using SPSS 29. Then, M*plus* 8.7 (Muthén & Muthén, 2017) was used to perform multilevel analyses. Two-level multilevel path analysis models were used to examine the daily associations between each pornography use motivation and same-day positive behaviors and between each pornography use motivation and same-day negative behaviors (i.e., 18 models in total). In each model, both partners’ scores were modeled as multivariate outcomes and residual terms were allowed to be correlated between partners. Dyads were considered indistinguishable as this sample included both same-gender and mixed-gender couples, therefore gender could not distinguish partners within all dyads. Daily reports (Level 1) were considered nested within couples (Level 2) with each partner randomly assigned to “partner 1” and “partner 2” and adding equality constraints on all parameters between partners. The analyses were guided by the actor-partner interdependence model (APIM; Kenny et al., 2006) which allowed to test both actor effects (e.g., association between one’s own pornography use motivation and own positive and negative behaviors) controlling for partner effects (e.g., association between one’s own pornography use motivation and their partner’s positive and negative behaviors) and partner effects controlling for actor effects. A random intercept and random slopes for the effects of pornography use motivations were estimated. As partners were indistinguishable, there were two slopes constraint to be equal for each actor effect, two slopes constraint to be equal for each partner effect, and two intercepts constraint to be equal. The multilevel analyses were performed with the robust maximum likelihood estimator (MLR) to take into account the naturally non-normal distribution of the data. On days both partners completed the diary, score-level missing data were handled using full information maximum likelihood (FIML). All coefficients reported are unstandardized coefficients and represent the change in the outcome variables (positive and negative behaviors) when using pornography for this motivation relative to days without pornography use for this motivation.

We controlled for linear time as Level-1 predictor (within-person effect) to account for the passage of time over the 35-day period. As level-2 predictors (between-person effects), we controlled for relationship duration, religiosity, overall pornography use motivation (i.e., total days a person used pornography for the given motivation over the diary period), stress related to COVID-19, masturbation frequency (i.e., total days a person masturbated), and partnered sexual activity frequency (i.e., total days couples reported sexual activity). These variables were chosen based on prior research: positive and negative behaviors may evolve over the course of a romantic relationship (e.g., less positive and more negative behaviors; Manning et al., 2018), religiosity has been linked to negative pornography use outcomes (Grubbs et al., 2019), controlling for overall pornography use motivation allows to isolate within-person associations in daily analyses, masturbation often occurs alongside pornography use and may be related more strongly to some motivations (Prause, 2019), and pornography use may follow unmet desire for partnered sexual activity or lead to partnered sexual activity (Vaillancourt-Morel et al., 2020). Given that data collection occurred during the COVID-19 pandemic, we also accounted for COVID-19-related stress.

To examine differences in the associations between pornography use motivations and positive and negative behaviors between cisgender men and cisgender women, cross-level interactions with the participant’s gender (0 = *cisgender man*; 1 = *cisgender woman*) was added in each model. When one interaction term was significant, simple slope tests were performed to report the association for cisgender men and cisgender women separately. Although efforts were made to include as many gender-diverse participants as possible, the limited sample size (*n* = 22) prevented us from including these participants in the gender difference analyses.

For the lagged-day analyses, we predicted next-day outcomes adjusting for the participant’s score today. Thus, the outcome represents the residualized change that occurred in this outcome since the prior day. For example, we examined whether today’s pornography use motivations were associated with next-day positive behaviors, controlling for today’s positive behaviors.

## Results

### Descriptive analyses

The 654 participants (*n* = 327 couples) individually completed a total of 19,735 diaries out of 22,890 (654 participants for 35 days) for a completion rate of 86.2% (*M* = 30.18 diaries out of 35, *SD* = 7.94). Same day diaries between partners were matched to form 10,519 couple entries in a dyadic dataset with 13.0% (1,370 days) of these entries for which only one partner completed the pornography use measure that day. For the APIM, data were only included for days when both partners provided a valid answer regarding their pornography use (i.e., yes, or no) leaving a total of 9,149 days across couples. If a person did not use pornography that day, a score of 0 was attributed to all motivations and the day was included in the analyses.

Means, standard deviations, and range for daily measures aggregated within-person across all diaries are shown in Table 1 for the total sample and in Supplemental Table S1 by gender. Participants reported using pornography an average of 4.00 days out of the 35 days, ranging from 0 to 34 days (*SD* = 5.81). Bivariate correlations for daily measures aggregated within-person across all diaries and control variables are shown in Table 1.

**Table 1.** Descriptive Statistics and Correlations Among Aggregated Daily Variables and Control Variables (*n* = 327 couples).

	1	2	3	4	5	6	7	8	9	10	11	12	13	14	15	16
1. Sexual pleasure PUM	**.06**	.05	.04	.03	−.01	.02	.06	.04	.15^***^	.01	.04	−.04	.11^**^	.003	.08*	-
2. Sexual curiosity PUM	.08^*^	**.12** ^**^	−.02	.02	−.02	.03	.03	.08	.06	.10^**^	<.001	−.02	−.03	−.002	.06	-
3. Fantasy PUM	.42^***^	.09^*^	**−.03**	−.01	−.02	−.04	−.01	.01	.07	−.05	.08^*^	.003	.07	.01	.06	-
4. Boredom avoidance PUM	.38^***^	.14^***^	.45^***^	**.18** ^***^	<.001	.02	.10*	.05	.16^***^	−.01	.03	−.02	.06	−.006	.03	-
5. Lack of sexual satisfaction PUM	.36^***^	.04	.41^***^	.11^**^	**−.02**	−.02	−.02	−.01	−.01	−.10^**^	.10^*^	.01	.11^**^	.03	.02	-
6. Emotional distraction PUM	.35^***^	.06	.43^***^	.47^***^	.21^***^	**−.01**	.02	.02	.15^***^	−.01	.10^*^	−.07	.10^*^	−.002	.06	-
7. Stress reduction PUM	.49^***^	.03	.49^***^	.46^***^	.48^***^	.69^***^	**.04**	.04	.16^***^	−.01	.09^*^	−.05	.13^**^	.03	.09^*^	-
8. Self-exploration PUM	.34^***^	.13^***^	.58^***^	.37^***^	.40^***^	.53^***^	.45^***^	**.04**	.12^**^	−.05	.18^***^	−.03	.05	−.03	.10^*^	-
9. Partner-related PUM	.22^***^	.08	.14^***^	.24^***^	−.002	.27^***^	.19^***^	.18^***^	**.43** ^***^	.06	.01	−.05	−.02	.06	.11^**^	-
10. Positive behaviors	−.05	.07	−.06	−.08^*^	−.13^***^	−.04	−.05	−.07	.07	**.61** ^***^	−.19^***^	−.22^***^	−.01	.10^*^	.02	-
11. Negative behaviors	−.03	.05	.05	.02	.08^*^	.08^*^	.05	.12^***^	.004	−.20^***^	**.48** ^***^	−.10^*^	.03	.07	.05	-
12. Relationship duration (years)	−.04	−.02	.01	−.02	.01	−.07	−.05	−.03	−.05	−.21^***^	−.10^*^	**.99** ^***^	−.001	.04	−.05	-
13. Stress related to COVID-19	−.05	.01	.03	.03	−.04	.12^**^	.02	−.003	−.04	.01	.16^***^	.002	**.27** ^***^	.04	.10^**^	-
14. Religiosity	−.02	.01	−.01	−.03	−.01	−.01	−.01	−.04	.07	.10^**^	.06	.04	.15^***^	**.51** ^***^	.01	-
15. Masturbation frequency	.78^***^	.16^***^	.34^***^	.33^***^	.29^***^	.34^***^	.47^***^	.32^***^	.16^***^	−.03	−.03	−.05	−.06	−.001	**.13** ^**^	-
16. Sexual activity frequency	.11^**^	.10^**^	.04	.09^*^	−.08^*^	.14^***^	.13^**^	.04	.27^***^	.36^***^	−.11^**^	−.19^***^	−.07	.05	.12^**^	-
Mean (*SD*)	3.18 (4.88)	0.08 (0.46)	0.49 (2.26)	0.75 (2.89)	0.35 (1.78)	0.36 (1.60)	1.00 (3.07)	0.50 (1.86)	0.09 (0.49)	4.60 (1.07)	1.20 (0.30)	6.32 (5.89)	3.54 (1.45)	4.33 (2.44)	5.01 (5.85)	6.80 (5.99)
Range	0–31	0–7	0–28	0–33	0–30	0–24	0–30	0–24	0–8	1.23–6.99	1.00–3.10	0.50–34.67	1–7	3–18	0–31	0–35

*Notes. SD* = Standard deviation; PUM = Pornography use motivation; Masturbation frequency = Total days a person masturbated; Sexual activity frequency = Total days couples reported sexual activity. Correlations below the diagonal are between each of the actor variables and correlations along (in bold) and above the diagonal are between the actor and partner variables.

*p < .05. **p < .01. ***p < .001.

### Daily associations between pornography use motivations and positive behaviors

Results for the associations between a person’s pornography use motivations and their own and their partner’s daily positive behaviors are presented in Table 2. Results showed that using pornography for sexual pleasure, fantasy, boredom avoidance, lack of sexual satisfaction, and self-exploration motivations were not significantly related to one’s own positive behaviors on that day but were significantly related to one’s partner’s lower positive behaviors on that day. Results also showed that using pornography for stress reduction and emotional distraction or suppression motivations were related to one’s own and the partner’s lower positive behaviors on that day. Moreover, using pornography for partner-related motivation was significantly related to one’s own higher positive behaviors on that day whereas it was not significantly related to the partner’s positive behaviors. Finally, using pornography for sexual curiosity motivation was not significantly related to one’s own or the partner’s positive behaviors on that day.

**Table 2.** Daily Associations Between Actor and Partner Pornography Use Motivations and Daily Positive Behaviors (*n* = 327 couples, 9149 days).

Fixed effects (intercept, slopes)	Positive behaviors				
	Estimate (*SE*)^a^	Z	*p*-value	95% CI	
				Lower	Upper
Sexual pleasure PUM					
Intercept	4.22 (.21)	20.49	<.001	3.82	4.63
Actor pornography use	−0.03 (.03)	−0.81	.418	−0.09	0.04
Partner pornography use	−0.14 (.04)	−3.80	<.001	−0.21	−0.07
Sexual curiosity PUM					
Intercept	4.22 (.21)	20.49	<.001	3.82	4.62
Actor pornography use	0.28 (.15)	1.89	.059	−0.01	0.57
Partner pornography use	0.05 (.15)	0.33	.742	−0.25	0.35
Fantasy PUM					
Intercept	4.18 (.21)	20.00	<.001	3.77	4.59
Actor pornography use	0.05 (.08)	0.67	.501	−0.10	0.20
Partner pornography use	−0.17 (.08)	−2.10	.035	−0.32	−0.01
Boredom avoidance PUM					
Intercept	4.20 (.21)	20.26	<.001	3.79	4.60
Actor pornography use	−0.02 (.06)	−0.24	.810	−0.14	0.11
Partner pornography use	−0.24 (.07)	−3.44	.001	−0.37	−0.10
Lack of sexual satisfaction PUM					
Intercept	4.21 (.21)	20.32	<.001	3.81	4.62
Actor pornography use	−0.18 (.09)	−1.93	.054	−0.36	0.003
Partner pornography use	−0.23 (.10)	−2.37	.018	−0.42	−0.04
Emotional distraction or suppression PUM					
Intercept	4.13 (.21)	19.48	<.001	3.72	4.55
Actor pornography use	−0.29 (.09)	−3.13	.002	−0.48	−0.11
Partner pornography use	−0.26 (.09)	−2.91	.004	−0.43	−0.09
Stress reduction PUM					
Intercept	4.16 (.21)	19.46	<.001	3.74	4.58
Actor pornography use	−0.23 (.06)	−3.69	<.001	−0.36	−0.11
Partner pornography use	−0.23 (.05)	−4.16	<.001	−0.33	−0.12
Self-exploration PUM					
Intercept	4.22 (.21)	20.51	<.001	3.81	4.62
Actor pornography use	−0.10 (.07)	−1.38	.169	−0.24	0.04
Partner pornography use	−0.21 (.08)	−2.61	.009	−0.37	−0.05
Partner-related PUM					
Intercept	4.20 (.21)	20.33	<.001	3.80	4.61
Actor pornography use	0.66 (.18)	3.68	<.001	0.31	1.01
Partner pornography use	0.35 (.26)	1.33	.183	−0.17	0.86

^a^*Notes.* Estimates are unstandardized regression coefficients; PUM = Pornography use motivation; *SE* = Standard error; Z = Estimate divided by standard error; CI = Confidence interval. Relationship duration, religiosity, the sum of days a person used pornography for the given motivation over the diary period, linear time, stress related to COVID-19, masturbation frequency, and partnered sexual activity frequency were included as control variables.

There were three significant interactions revealing significant differences between cisgender men and cisgender women in the strength of these associations. The association between a person’s pornography use for sexual pleasure and the partner’s daily positive behaviors was significantly different between women and men (*b* = 0.18, *SE* = .08; *p* = .015). Simple slopes indicated that on days cisgender men used pornography for sexual pleasure, their partner reported lower positive behaviors on that day (*b* = −0.20, *SE* = .04, *p* < .001), whereas for cisgender women, pornography use for sexual pleasure motivation was unrelated to their partner’s positive behaviors (*b* = −0.01, *SE *= .07, *p* = .830). The association between a person’s pornography use for partner-related motivation and their own’s (*b* = −0.20, *SE* = .01, *p* < .001) and the partner’s daily positive behaviors (*b* = 0.65, *SE* = .32, *p* = .042) were also significantly different between women and men. Simple slopes indicated that on days cisgender men or cisgender women used pornography for partner-related motivation, they reported higher positive behaviors toward their partner on that day, but the association was significantly stronger for men (men: *b* = 0.76, *SE* = .19, *p* < .001; women: *b* = 0.56, *SE* = .23, *p* = .014). Finally, on days cisgender women used pornography for partner-related motivation, their partner reported higher positive behaviors on that day (*b* = 0.80, *SE* = .30, *p* = .008), whereas for cisgender men, pornography use for partner-related motivation was unrelated to their partner’s positive behaviors (*b* = 0.15, *SE* = .25, *p* = .551).

### Daily associations between pornography use motivations and negative behaviors

Results for the associations between a person’s pornography use motivations and their own and their partner’s daily negative behaviors are presented in Table 3. Results showed that using pornography for sexual pleasure motivation was related to one’s own lower negative behaviors on that day, whereas it was not significantly related to one’s partner’s negative behaviors. Moreover, using pornography for sexual curiosity motivation was not significantly related to one’s own negative behaviors on that day, whereas it was related to the partner’s lower negative behaviors. Also, using pornography for emotional distraction or suppression motivation was related to one’s own higher negative behaviors on that day whereas it was not significantly related to the partner’s negative behaviors. Finally, using pornography for fantasy, boredom avoidance, lack of sexual satisfaction, stress reduction, self-exploration, and partner-related motivations were not significantly related to one’s own or to the partner’s negative behaviors on that day. There were no significant differences between cisgender men and cisgender women in these associations.

**Table 3.** Daily Associations Between Actor and Partner Pornography Use Motivations and Daily Negative Behaviors (*n* = 327 couples, 9149 days).

Fixed effects (intercept, slopes)	Negative behaviors				
				95% CI	
	Estimate (*SE*)^a^	Z	*p*-value	Lower	Upper
Sexual pleasure PUM					
Intercept	1.17 (.06)	21.15	<.001	1.06	1.28
Actor pornography use	−0.03 (.01)	−2.24	.025	−0.06	−0.004
Partner pornography use	−0.02 (.01)	−1.55	.122	−0.05	0.006
Sexual curiosity PUM					
Intercept	1.17 (.06)	21.43	<.001	1.06	1.28
Actor pornography use	−0.03 (.05)	−0.71	.477	−0.13	0.06
Partner pornography use	−0.09 (.04)	−2.61	.009	−0.16	−0.02
Fantasy PUM					
Intercept	1.18 (.06)	21.48	<.001	1.08	1.29
Actor pornography use	−0.04 (.03)	−1.30	.193	−0.09	0.02
Partner pornography use	−0.004 (.07)	−0.06	.951	−0.14	0.13
Boredom avoidance PUM					
Intercept	1.17 (.06)	21.39	<.001	1.07	1.28
Actor pornography use	−0.02 (.03)	−0.52	.604	−0.08	0.05
Partner pornography use	−0.01 (.04)	−0.28	.781	−0.08	0.06
Lack of sexual satisfaction PUM					
Intercept	1.17 (.06)	21.37	<.001	1.06	1.28
Actor pornography use	0.04 (.04)	0.86	.390	−0.05	0.12
Partner pornography use	0.06 (.08)	0.70	.482	−0.10	0.21
Emotional distraction or suppression PUM					
Intercept	1.20 (.05)	22.21	<.001	1.09	1.30
Actor pornography use	0.12 (.05)	2.55	.011	0.03	0.21
Partner pornography use	0.15 (.09)	1.81	.070	−0.01	0.32
Stress reduction PUM					
Intercept	1.19 (.06)	20.83	<.001	1.08	1.30
Actor pornography use	<0.001 (.03)	0.003	.998	−0.07	0.07
Partner pornography use	0.002 (.04)	0.05	.963	−0.08	0.09
Self-exploration PUM					
Intercept	1.17 (.05)	21.77	<.001	1.07	1.28
Actor pornography use	−0.02 (.04)	−0.65	.516	−0.09	0.05
Partner pornography use	0.02 (.05)	0.48	.631	−0.07	0.12
Partner-related PUM					
Intercept	1.17 (.06)	21.41	<.001	1.07	1.28
Actor pornography use	0.10 (.12)	0.87	.383	−0.13	0.34
Partner pornography use	−0.03 (.04)	−0.68	.495	−0.10	0.05

^a^*Notes.* Estimates are unstandardized regression coefficients; PUM = Pornography use motivation; *SE* = Standard error; Z = Estimate divided by standard error; CI = Confidence interval. Relationship duration, religiosity, the sum of days a person used pornography for the given motivation over the diary period, linear time, stress related to COVID-19, masturbation frequency, and partnered sexual activity frequency were included as control variables.

### Directionality of daily associations

We conducted lagged-day analyses to examine whether the associations found concurrently on the same day persisted into the next day. Results are presented in Supplemental Table S2 for positive behaviors and Supplemental Table S3 for negative behaviors. Results showed that neither a participant’s pornography use motivations today nor their partner’s pornography use motivations today were significantly associated with next-day positive or negative behaviors, after controlling for their own positive and negative behaviors today. These results suggest that pornography use motivations and positive and negative behaviors occurred concurrently, within the same time frame, but are not predictive of next-day behaviors.

## Discussion

The present study used a dyadic daily diary design to examine same-day and next-day associations between pornography use motivations and positive and negative behaviors between partners in adult couples from the community. Findings indicated that on days when a person used pornography to reduce stress, they reported fewer positive behaviors toward their partner and, when they used pornography to distract or suppress their emotions, they reported fewer positive behaviors and greater negative behaviors toward their partner. However, when a person used pornography as a sexual activity with their partner, they reported more positive behaviors toward their partner that day and when they used pornography for sexual pleasure, they had fewer negative behaviors that day. Partner effects indicated that when a person used pornography to stimulate fantasy, to avoid boredom, because they are sexually dissatisfied, to supress or distract from painful emotional states, to reduce stress, and to explore what arouses them sexually, their partner reported fewer positive behaviors that day. Moreover, on days cisgender men used pornography for sexual pleasure, their partner reported lower positive behaviors that day, and when a person used pornography out of curiosity, their partner reported fewer negative behaviors that day. However, on days cisgender women used pornography for partner-related motivation, their partner reported higher positive behaviors on that day. Findings extend and support the ACE model and show the importance of taking into consideration the context underlying pornography use as motivations for this sexual behavior are related to daily couple behaviors (Campbell & Kohut, 2017).

### Daily associations between pornography use motivations and positive behaviors

In line with our hypotheses, on days a person used pornography for partner-related motivation (approach motivation), they reported expressing greater positive behaviors toward their partner the same day. This result is in line with previous studies showing that using pornography with the partner is associated with positive relational outcomes, including greater sexual and emotional intimacy and sexual satisfaction (Huntington et al., 2021; Willoughby & Leonhardt, 2020). Thus, when partners use pornography together, they might feel closer to each other, and therefore their interactions that day may be more positive. Moreover, on days a person used pornography for stress reduction and emotional distraction or suppression (avoidance motivations), they reported expressing fewer positive behaviors toward their partner. Findings are in line with the approach-avoidance sexual motivations theory (Impett et al., 2005). Moreover, they support the only past study focusing on pornography use motivations in couples (Bőthe et al., 2022) whereby approach sex motives were related to positive outcomes (e.g., greater relationship satisfaction) and avoidance sex motives were associated with negative outcomes (e.g., greater conflicts, lower sexual function, and greater sexual distress). When a person uses pornography to cope with their emotions and stress, it may represent a less adaptive emotion regulation strategy and be related with negative relational outcomes (i.e., less positive behaviors).

Our findings also indicated that on days a person used pornography for approach motivations (i.e., sexual pleasure and self-exploration) and avoidance motivations (i.e., fantasy, boredom avoidance, lack of sexual satisfaction, emotional distraction or suppression, and stress reduction), their partner reported expressing fewer positive behaviors toward them. Overall, regardless of the motivations, except for sexual curiosity and partner-related motivations, when a person used pornography, their partner reported fewer positive behaviors on the same day. This result is in line with previous studies on pornography use frequency which showed that use was related to negative outcomes for the partner, including lower intimacy and relationship satisfaction (Vaillancourt-Morel et al., 2023; Willoughby & Leonhardt, 2020). Even when a person knows about their partner’s pornography use, they might not know the motivations underlying this use (approach or avoidance motivations), which might explain that they expressed fewer positive behaviors toward their partner that day regardless of the motivation. Moreover, on days cisgender men used pornography for sexual pleasure, their partner reported lower positive behaviors that day, whereas for cisgender women, this motivation was unrelated to their partner’s positive behaviors. This result is also in line with previous studies on pornography use and relational and sexual wellbeing showing that men’s pornography use is associated with their partner’s negative outcomes, including lower intimacy and sexual desire (Vaillancourt-Morel et al., 2023; Willoughby & Leonhardt, 2020). In our sample, where most men were partnered with a woman, one possible explanation is that women may perceived their men partner’s pornography use negatively due to the objectification and the degradation of women in pornographic content (Sun et al., 2016). As a result, women partners may be less inclined to express positive behaviors toward their partner on the same day. Finally, on days cisgender women used pornography for partner-related motivation, their partner reported higher positive behaviors on that day. This finding aligns with previous research suggesting that women’s pornography use is related to their partner’s positive outcomes, including, higher sexual desire and higher odds of partnered sexual activity (Vaillancourt-Morel et al., 2020). Given that women’s partnered pornography use has been linked to greater sexual function and lower sexual distress (Bőthe et al., 2022), it is possible that when women use pornography for partner-related motivation, it is experienced as pleasurable prompting the partner to express more positive behaviors on those days.

### Daily associations between pornography use motivations and negative behaviors

In line with our hypotheses, on days a person used pornography for sexual pleasure (approach motivation), they reported expressing fewer negative behaviors toward their partner the same day, whereas on days a person used pornography for emotional distraction or suppression (avoidance motivations), they reported expressing greater negative behaviors toward their partner. These results are in line with a previous study in which approach motivations were related to positive outcomes including higher partnered sexual frequency and sexual satisfaction, whereas avoidance motivations were associated with negative outcomes including higher sexual distress (Bőthe et al., 2022). Moreover, as using pornography for emotion regulation is related to problematic pornography use (Bőthe et al., 2024), this compulsive use may in turn lead to conflictual interactions that day, which may explain that the person using pornography expressed higher negative behaviors.

Our findings also indicated that on days a person used pornography for sexual curiosity (approach motivation), their partner reported expressing fewer negative behaviors toward them. When a person uses pornography out of curiosity, it might improve their sexual life the same day (e.g., learn and apply new sexual position). Therefore, the partner might appreciate the initiative of the other to bring something new in their sexual life which in turn, lead the partner to express fewer negative behaviors that day.

### Implications for practice

Our results could help therapists working with couples to better understand the interplay between couple dynamics and pornography use. Therapists should identify not only partners’ frequency of pornography use, but their pornography use motivations as well. Indeed, our findings showed that pornography use motivations are related to daily couple interactions as they are linked to partners’ positive and negative behaviors and may fuel positive, or less positive, couple dynamics. Also, as avoidance motivations related to emotional regulation may reflect more general underlying difficulties in coping with negative emotions and stress, promoting more adaptive emotion regulation strategies may be beneficial to daily couple interactions (Bőthe et al., 2022).

### Limitations and futures studies

Some limitations should be considered when interpreting the results. First, the daily diaries design and the lack of statistical control for other potential confounding factors makes it impossible to determine causal relations. Although, we examined the associations with next-day behaviors, the results suggest these associations operate within the same day, providing no insight into directionality. Convergence issues further prevent testing the directionality between pornography use motivations and positive and negative behaviors across days. Second, even if we used daily diaries to minimize recall biases, self-report measures, specifically on sensitive subjects such as pornography use and expression of negative behaviors toward the partner, are subject to under- or over-reporting due to social desirability. Third, the findings’ generalizability is limited by our non-representative convenience sample of adult couples from the community where self-selection biases may occur. Our sample was composed mainly of White participants, with high educational degrees, as well as high personal incomes, and included only 22 gender diverse individuals. Future studies should oversample sexual and gender diverse participants and individuals from various ethnic groups and include individuals from a larger range of socio-economic backgrounds. Moreover, future studies should consider that different relational contexts surrounding pornography use (e.g., secret use), and individual characteristics (e.g., emotion regulation strategies, sexual desire) may affect the associations between pornography use and daily couple dynamics. Indeed, we did not assess whether sexual activity occurred before or after pornography use, nor whether a partner initiated sexual activity and was rejected. These variables could provide valuable insight into the complex interplay between partnered sexual activity and motivations for pornography use. Finally, future studies should examine more closely the mechanisms underlying the associations between pornography use motivations and positive and negative behaviors.

### Conclusion

In line with the approach-avoidance sexual motivations theory (Impett et al., 2005), our results show that when a person used pornography for avoidance motivations related to emotional regulation (e.g., emotional distraction or suppression and stress reduction motivations), it was associated negatively with the couple interactions (i.e., fewer positive and higher negative behaviors). Overall, independently of a person’s approach or avoidance motivations for using pornography, a person’s pornography use was related to their partner reporting fewer positive behaviors on the same day. Conversely, some approach motivations (e.g., sexual pleasure, sexual curiosity, and partner-related motivations) were related positively to the couple dynamic (i.e., higher positive and fewer negative behaviors). Findings that pornography use motivations are related to couples’ daily behaviors highlight the importance of taking into consideration the context behind pornography use in research and practice.

## Supplemental Material

Supplemental Material - Are pornography use motivations related to behaviors toward the romantic partner? A dyadic daily diary study Supplemental Material for Are pornography use motivations related to behaviors toward the romantic partner? A dyadic daily diary study by Mandy Vasquez, Marie-Ève Daspe, Beáta Bőthe, Sophie Bergeron, Samantha J. Dawson and Marie-Pier Vaillancourt-Morel in Journal of Social and Personal Relationships
