# Supplementary material for: Are pornography use motivations related to behaviors toward the romantic partner? A dyadic daily diary study
Source: J Soc Pers Relat. 2025 Apr 22;42(8):2078–98. doi: 10.1177/02654075251335813 (PMC12176280; doi:10.1177/02654075251335813)
Supplement: Supplemental Material - Are pornography use motivations related to behaviors toward the romantic partner? A dyadic daily diary study [file sj-pdf-1-spr-10.1177_02654075251335813.pdf]

Supplemental Material

Table S1

	Cisgender men (*n* = 298)		Cisgender women (*n* = 330)		Gender diverse individuals (*n* = 22)	
	M (SD)	Observed Range	*M (SD)*	Observed Range	*M (SD)*	Observed range
Sexual pleasure PUM	4.76 (6.14)	0-31	1.64 (2.55)	0-15	4.45 (4.32)	0-13
Sexual curiosity PUM	0.08 (0.52)	0-7	0.08 (0.41)	0-4	0.09 (0.29)	0-1
Fantasy PUM	0.74 (3.14)	0-28	0.26 (1.03)	0-9	0.36 (0.79)	0-3
Boredom avoidance PUM	1.18 (3.89)	0-33	0.32 (1.31)	0-13	0.77 (1.90)	0-6
Lack of sexual satisfaction PUM	0.59 (2.48)	0-30	0.14 (0.78)	0-11	0.32 (0.78)	0-3
Emotional distraction or suppression PUM	0.49 (1.95)	0-24	0.16 (0.80)	0-9	0.86 (1.98)	0-8
Stress reduction PUM	1.42 (3.85)	0-30	0.55 (1.95)	0-23	1.32 (2.19)	0-7
Self-exploration PUM	0.70 (2.36)	0-24	0.27 (0.83)	0-7	1.32 (4.00)	0-19
Partner-related PUM	0.09 (0.44)	0-4	0.08 (0.53)	0-8	0.14 (0.47)	0-2
Positive behaviors	4.52 (1.08)	1.36-6.94	4.63 (1.07)	1.23-6.99	5.09 (0.89)	3.87-6.88
Negative behaviors	1.16 (0.29)	1.00-3.10	1.23 (0.31)	1.00-3.03	1.23 (0.32)	1.00-2.20

Aggregated Pornography Use Motivations, Positive and Negative Behaviors by Gender (n = 327 couples)

Notes. M = Mean; *SD* = Standard deviation; PUM = Pornography use motivation. Gender diverse individuals are participants reporting a non-binary, genderfluid, multi-gender, genderqueer, agender, or another gender identity.

Table S2

Lagged-Day Analyses Between Today Actor and Partner Pornography Use Motivations and Next Day Positive Behaviors, Controlling for Today Positive Behaviors (n = 327 couples, 9149 days)

	Next day positive behaviors				
Fixed effects (intercept, slopes)	Estimate (*SE*)[a]	Z	*p*-value	95% CI Lower	95% CI Upper
Today sexual pleasure PUM					
Intercept	3.67 (.09)	42.89	< .001	3.53	3.87
Actor pornography use	-0.03 (.03)	-0.89	.375	-0.09	0.03
Partner pornography use	-0.004 (.03)	-0.15	.883	-0.06	0.06
Today positive behaviors	0.20 (.01)	16.04	< .001	0.18	0.23
Today sexual curiosity PUM					
Intercept	3.65 (.08)	48.66	< .001	3.50	3.79
Actor pornography use	0.14 (.19)	0.73	.463	-0.24	0.52
Partner pornography use	0.19 (.15)	1.27	.203	-0.10	0.47
Today positive behaviors	0.20 (.01)	15.96	< .001	0.18	0.23
Today fantasy PUM					
Intercept	3.70 (.08)	48.56	< .001	3.55	3.84
Actor pornography use	0.02 (.08)	0.18	.855	-0.15	0.18
Partner pornography use	-0.04 (.08)	-0.48	.631	-0.19	0.12
Today positive behaviors	0.20 (.01)	16.02	< .001	0.18	0.23
Today boredom avoidance PUM					
Intercept	3.69 (.08)	48.69	< .001	3.54	3.84
Actor pornography use	0.01 (.21)	0.04	.971	-0.39	0.41
Partner pornography use	-0.04 (.11)	-0.40	.692	-0.26	0.17
Today positive behaviors	0.20 (.01)	15.84	< .001	0.18	0.23
Today lack of sexual satisfaction PUM					
Intercept	3.71 (.08)	48.39	< .001	3.56	3.86
Actor pornography use	-0.08 (.11)	-0.69	.492	-0.30	0.15
Partner pornography use	0.01 (.12)	0.08	.934	-0.23	0.25
Today positive behaviors	0.20 (.01)	16.04	< .001	0.18	0.23
Today emotional distraction or suppression PUM					
Intercept	3.68 (.08)	48.13	< .001	3.53	3.83
Actor pornography use	0.002 (.12)	0.02	.985	-0.22	0.23
Partner pornography use	0.05 (.09)	0.63	.528	-0.11	0.22
Today positive behaviors	0.20 (.01)	15.97	< .001	0.18	0.23
Today stress reduction PUM					
Intercept	3.69 (.08)	47.62	< .001	3.54	3.84
Actor pornography use	0.04 (.06)	0.62	.533	-0.09	0.17
Partner pornography use	0.04 (.06)	0.59	.533	-0.08	0.16
Today positive behaviors	0.20 (.01)	15.98	< .001	0.18	0.23
Today self-exploration PUM					
Intercept	3.70 (.08)	48.49	< .001	3.55	3.85
Actor pornography use	-0.04 (.09)	-0.46	.647	-0.22	0.14
Partner pornography use	-0.04 (.08)	-0.45	.653	-0.20	0.12
Today positive behaviors	0.20 (.01)	16.09	< .001	0.18	0.23
Today partner-related PUM					
Intercept	3.66 (.07)	49.17	< .001	3.51	3.80
Actor pornography use	-0.08 (.16)	-0.48	.633	-0.39	0.24
Partner pornography use	0.16 (.19)	0.83	.408	-0.22	0.53
Today positive behaviors	0.20 (.01)	15.92	< .001	0.18	0.23

Notes. [a] = Estimates are unstandardized regression coefficients; PUM = Pornography use motivation; *SE* = Standard error; Z = Estimate divided by standard error; CI = Confidence interval.

	Next day negative behaviors	
		95% CI

Table S3

Fixed effects (intercept, slopes)	Estimate (*SE*)[a]	Z	*p*-value	Lower	Upper
Today sexual pleasure PUM					
Intercept	1.02 (.03)	36.44	< .001	0.96	1.07
Actor pornography use	0.01 (.02)	0.75	.453	-0.02	0.04
Partner pornography use	0.01 (.01)	0.96	.336	-0.01	0.04
Today negative behaviors	0.14 (.02)	7.75	< .001	0.11	0.18
Today sexual curiosity PUM					
Intercept	1.01 (.02)	41.90	< .001	0.97	1.06
Actor pornography use	-0.01 (.08)	-0.11	.916	-0.16	0.14
Partner pornography use	-0.07 (.08)	-0.89	.373	-0.24	0.09
Today negative behaviors	0.14 (.02)	7.63	< .001	0.10	0.18
Today fantasy PUM					
Intercept	1.01 (.02)	42.57	< .001	0.96	1.06
Actor pornography use	0.07 (.05)	1.29	.196	-0.03	0.16
Partner pornography use	0.02 (.04)	0.67	.501	-0.04	0.09
Today negative behaviors	0.14 (.02)	7.68	< .001	0.11	0.18
Today boredom avoidance PUM					
Intercept	1.02 (.02)	42.95	< .001	0.97	1.06
Actor pornography use	0.003 (.03)	0.10	.918	-0.05	0.06
Partner pornography use	0.02 (.04)	0.58	.564	-0.06	0.10
Today negative behaviors	0.14 (.02)	7.69	< .001	0.11	0.18
Today lack of sexual satisfaction PUM					
Intercept	1.01 (.02)	42.43	< .001	0.96	1.05
Actor pornography use	0.01 (.06)	0.23	.821	-0.10	0.12
Partner pornography use	0.02 (.06)	0.30	.762	-0.10	0.14
Today negative behaviors	0.14 (.02)	7.79	< .001	0.11	0.18
Today emotional distraction or suppression PUM					
Intercept	1.01 (.02)	44.29	< .001	0.97	1.05
Actor pornography use	0.03 (.04)	0.70	.482	-0.05	0.10
Partner pornography use	-0.002 (.05)	-0.05	.962	-0.10	0.09
Today negative behaviors	0.14 (.02)	7.62	< .001	0.11	0.18
Today stress reduction PUM					
Intercept	1.01 (.02)	41.46	< .001	0.96	1.06
Actor pornography use	0.01 (.03)	0.38	.706	-0.05	0.07
Partner pornography use	-0.01 (.03)	-0.28	.783	-0.06	0.05
Today negative behaviors	0.14 (.02)	7.71	< .001	0.11	0.18
Today self-exploration PUM					
Intercept	1.00 (.02)	42.99	< .001	0.95	1.05
Actor pornography use	0.05 (.05)	1.00	.317	-0.04	0.13
Partner pornography use	0.02 (.03)	0.60	.546	-0.04	0.08
Today negative behaviors	0.14 (.02)	7.67	< .001	0.11	0.18
Today partner-related PUM					
Intercept	1.02 (.02)	41.87	< .001	0.97	1.06
Actor pornography use	0.06 (.14)	0.38	.703	-0.23	0.34
Partner pornography use	0.06 (.17)	0.36	.720	-0.28	0.40
Today negative behaviors	0.14 (.02)	7.77	< .001	0.11	0.18

Lagged-Day Analyses Between Today Actor and Partner Pornography Use Motivations and Next Day Negative Behaviors, Controlling for Today Negative Behaviors (n = 327 couples, 9149 days)

Notes. [a] = Estimates are unstandardized regression coefficients; PUM = Pornography use motivation; *SE* = Standard error; Z = Estimate divided by standard error; CI = Confidence interval.
